# RISKY WORK ENVIRONMENTS AND METABOLIC DISEASE: CUMULATIVE CONSEQUENCES OF BAD JOBS IN EARLY AND MID LIFE

**DOI:** 10.1093/geroni/igad104.0436

**Published:** 2023-12-21

**Authors:** Rebekah Carpenter

**Affiliations:** Florida State University, Tallahassee, Florida, United States

## Abstract

Approximately one in three adults in the United States have a metabolic disease or illness. Metabolic diseases and illnesses are particularly costly for older adults, often resulting from decreased human capital and increased health care-based costs in later life. Social contexts have been hypothesized to be a key contributor to later-life health; however, work environments are often understudied as a critical social factor shaping health over the life course. Utilizing new data from the Health and Retirement Study’s Life History Mail Survey linked to occupational work context data from the Occupational Information Network (O*NET), we examine how exposure to dangerous work environments over one’s working career relates to the likelihood of developing a metabolic disease or illness between the ages of 50 and 62. Results from logistic regressions suggest that dangerous work environment exposures, such as exposures to contaminants or hazardous conditions, are associated with increased risk of developing metabolic disease and illnesses between ages 50 and 62; however, these associations are dependent upon when in the life course people are exposed to dangerous work contexts and for how long people are exposed to dangerous work contexts. This study speaks to the growing need for understanding the role of occupations as an important social and contextual factor shaping population health. Specifically, findings from this study may better inform the development of social and organizational policies targeted at improving work environments in the US, thereby helping to reduce the risk of developing health-related challenges, including metabolic diseases and illnesses.

